# A Narrative Review: Nutrition in Pediatric Nephrology: From Early Kidney Health to Advanced CKD Care

**DOI:** 10.3390/nu18213691

**Published:** 2026-11-07

**Authors:** Mirjam Močnik, Nataša Marčun Varda

**Affiliations:** 1 Department of Paediatrics, University Medical Centre Maribor, Ljubljanska Ulica 5, 2000 Maribor, Slovenia; natasa.marcunvarda@siol.net; 2 Medical Faculty, University of Maribor, Taborska 8, 2000 Maribor, Slovenia

**Keywords:** chronic kidney disease, child, malnutrition, protein-energy wasting, obesity, management

## Abstract

Children with chronic kidney disease require special nutritional care to ensure optimal growth and development. In children with declining kidney function, failure to thrive is a common problem. There is no universal diet that can be prescribed for these patients, as the nutritional plan must be adapted to the changing needs of the growing child and the stage and progression of chronic kidney disease. We must pay particular attention to energy and protein intake, but, due to various disease states, we also monitor intake of sodium, potassium, calcium, phosphorus, and iron. Achieving a balance between nutrients be a major challenge due to numerous dietary restrictions in children requiring renal replacement therapy. With appropriate nutritional planning and counseling, we can promote optimal growth and development in a child with chronic kidney disease. Besides failure to thrive, obesity as a persisting problem worldwide affects children with chronic kidney disease of all stages, leading to obesity-related renal injurious effects. Both undernutrition and overnutrition are significant challenges in children with chronic kidney disease, and nutritional management encompasses a wide range of measures, from preventing and treating obesity to providing a supportive diet and ensuring adequate protein and energy intake for weight gain and development. Here, we provide a narrative review summarizing pediatric nutritional management, covering topics from early kidney health to advanced chronic kidney disease care, and incorporating the latest findings as well as our own perspectives.

## 1. Introduction

Nutrition demands of children with chronic kidney disease (CKD) vary from those with obesity-related renal effects even in a healthy kidney [1] to protein-energy wasting in advanced CKD [2]. CKD in children is defined as structural or functional kidney damage persisting for at least 3 months, as evidenced by decreased glomerular filtration rate (GFR) stages 1–5, structural abnormalities on imaging or biopsy, or urinary markers, such as persistent hematuria or proteinuria [3]. Therefore, the spectrum of children with CKD is very wide. As obesity is on the rise globally [4], many children with CKD and preserved kidney function are at risk of developing excess weight, requiring a completely different approach to nutritional management in comparison to children with advanced CKD and lowered GFR, in whom failure to thrive is a very common challenge [5]; however, obesity and metabolic syndrome affect children at all CKD stages, including dialysis and children after renal transplantation [6]. Estimates of nutritional disorders in children with CKD were 20–45% for undernutrition and 15–30% for overnutrition in one single-center older study [7], and 25% for underweight and 6% for overweight in a more recent but again a single-center study with a low number of participants [8]. A more recent study with a large number of children with CKD included a report of 3% underweight, 65% normal weight, 15% overweight, and 16% obese patients at baseline [9].

Nutritional assessment should be a basic part of the evaluation of a child with CKD. There is no universal diet that can be prescribed for children with CKD. The term “renal diet” is sometimes used, but it is misleading, as the diet must be optimized and tailored to each patient. Nutritional therapy must be tailored to the changing needs of the growing child (age, current nutritional status, and growth parameters) and to the stage and progression of CKD.

In the nutritional management of children with CKD, a holistic and individualized approach is crucial, considering the child’s age, degree of renal impairment, nutritional status, growth and development, dietary habits, and family needs. A renal nutrition-focused dietitian plays a central role in this: planning, monitoring, and adapting nutritional management together with the child, parents, and the multidisciplinary health team. Their expertise enables timely identification of nutritional risks and the ensuring of adequate energy and nutrient intake, while supporting optimal growth, development, and quality of life for the child [10,11]. The Pediatric Renal Nutrition Task Force (PRNT), an international team of pediatric renal dietitians and pediatric nephrologists, was established to develop clinical practice recommendations to address these challenges and to serve as a resource for nutritional care [10,11].

While pediatric nephrologists and dietitians are crucial in nutritional management of a child with CKD, other medical professionals constitute a multidisciplinary team that altogether aim to provide nutritional support, such as nurses, a psychologist or behavioral specialist, a physiotherapist, and a social worker, providing a holistic approach to the management of children with advanced CKD, especially those who require renal replacement therapy [11,12].

Along with the multidisciplinary team, dietary management is strongly influenced by parents/caregivers, food availability, socioeconomic circumstances, cultural dietary customs, knowledge of food composition, and access to specialized renal dietetic care, which can significantly affect the nutritional status of a child with advanced CKD, again emphasizing the need for a multidisciplinary approach and a search for reasons for nutritional challenges not solely in the patients, but also in their family and environment [11,12]. This narrative review summarizes current evidence on nutrition in pediatric nephrology, covering topics from early kidney health to advanced CKD care.

## 2. Literature Overview

A narrative literature search was performed mainly in PubMed/MEDLINE, EMBASE, and the Cochrane Library using the following search terms: (‘chronic kidney disease’ OR ‘kidney health’) AND (‘children’) AND (‘nutrition’ OR ‘diet’ OR ‘obesity’ OR ‘undernutrition’ OR ‘nutritional demands’ OR ‘nutrition guidelines’) AND ‘children’. The search covered publications from January 2000 to July 2026, mainly to add recent guidelines provided by the PRNT. Additional references have been hand-searched as needed during the development of the manuscript. Given the narrative nature of this review, no formal PRISMA protocol was applied; however, priority was given to prospective studies, systematic reviews, meta-analyses, and pediatric-specific datasets.

## 3. Nutritional Assessment in Children with CKD

In infants and children under 2 years of age, body weight, body length, and head circumference are monitored. In patients older than 2 years, body weight, body height, body mass index (BMI), and growth velocity are monitored. Measurements are assessed based on appropriate growth curves. Age must be adjusted when determining values for premature infants. Growth parameters in children with CKD are monitored at least twice as frequently as in healthy children of the same age [13,14]. To be more specific, a recommendation is to monitor anthropometry every 3 months for children with CKD stages 2–4, and monthly for stage 5 [5]. Anthropometric measures are used to define nutritional status and differentiate children as underweight or overweight [5].

Nutritional status is also monitored by measuring biochemical parameters, including serum albumin, total protein, transferrin, and serum creatinine, as well as hemoglobin, total lymphocyte count, cholesterol, triglycerides, and retinol-binding protein. Although each of the above indicators contributes to a comprehensive assessment of nutritional status, none can serve as a sole marker, as it can be influenced by inflammation, hydration, kidney function, muscle mass, and other disease-related factors [14].

Similarly, PRNT includes anthropometric, dietary, and biochemical assessments as integral parts of nutritional assessment in children with CKD, interpreted longitudinally and in the context of kidney disease and fluid status [15]. Their recommendations are summarized in Table 1 [15].

Because children with CKD often have reduced muscle mass and increased fat mass [16], we use bioelectrical impedance analysis, dual-energy X-ray absorptiometry, or another method (usually more costly, such as computed tomography or magnetic resonance imaging) for additional body composition analysis. Body composition measurement can differentiate among body cell mass, fat mass, and total water, which helps with hydration and dry body weight evaluation [17,18,19]. Body composition assessment is also valuable for evaluating excess weight and determining fat mass, which can be more accurate for cardiovascular risk assessment than BMI alone [20]. Ultrasonography of muscles, especially the quadriceps, is gaining attention for detecting protein-energy wasting [5] and obesity-related sarcopenia, the latter mainly in the adult population; meanwhile, the utility in the pediatric clinical setting is still being researched [21].

After assessing nutritional status, we create an individualized nutritional plan to ensure adequate nutrient intake, promote growth and development, and provide dietary interventions for excess weight, possibly with renal dietitian [18,22]. Dietary intake is most often monitored using a three-day food diary or by recalling the previous day’s menu [18], especially in children aged 4–10 years; otherwise, a dietary recording of food intake over a week can provide more details. Also, a food-frequency questionnaire can be of help when a specific nutrient is in question [5,23]. Diet assessment also includes diet quality, appetite, taste and gastrointestinal symptoms [5].

## 4. Nutrition and Early Kidney Health in Children

There is no single diet specifically recommended to support kidney health. Instead, a healthy lifestyle approach and a balanced diet rich in fruits, vegetables, whole grains, legumes, nuts, and lean protein are recommended to support healthy blood pressure, weight, and metabolic health, which indirectly protects kidney function by maintaining a healthy cardiometabolic state [24]. Excess sodium can contribute to elevated blood pressure and increase the overall risk of kidney damage later in life; therefore, it is recommended to limit salt intake, as presented later in the article, with an emphasis on processed and packaged foods, which are sources of increased salt [23]. Also, these foods, along with sugar-sweetened beverages, are important sources of glucose, which should be limited when aiming for kidney health, as they help maintain healthy body weight and reduce cardiometabolic risk factors associated with potential future CKD. Along with that notion, encouraging healthy hydration by choosing water as the main beverage is important for kidney health, as it does not increase obesity risk and provides adequate kidney hydration and perfusion even in clinical conditions that could lead to pre-renal kidney injury due to hypovolemia [24]. Overall, kidney health in children is best supported by a healthy lifestyle that aims to maintain a healthy body weight and avoid obesity-associated risks of hypertension, diabetes, and chronic kidney disease [24]. Healthy children require protein for growth, but excess protein is not recommended as it has not been shown to improve kidney health [10].

## 5. Nutrition in Pediatric Chronic Kidney Disease and Overnutrition

While children with chronic kidney disease are traditionally characterized as being at risk for undernutrition, the nutritional status in patients varies and depends on the progression of kidney disease, other extrarenal conditions, genetics, overall dietary habits, and physical activity. Additionally, particular attention is directed to periods of rapid growth (infancy, puberty), during which nutrient requirements increase, also in children with CKD [11]. As long as the kidney function is preserved to the point where renal replacement therapy is not needed, the main recommendations state that the initial prescription for energy intake should approximate that of healthy children of the same chronological age and should be adjusted towards the higher end in those with suboptimal weight gain and linear growth [10]. Dietary protein restriction should be avoided as it may lead to malnutrition and protein-energy wasting. In addition, in recent years, an emphasis on incorporating plant-based protein foods has been encouraged, as they provide an important source of fiber [11], demonstrating a beneficial effect on obesity management in adolescents by preventing obesity-related complications [25].

**Figure 1:**
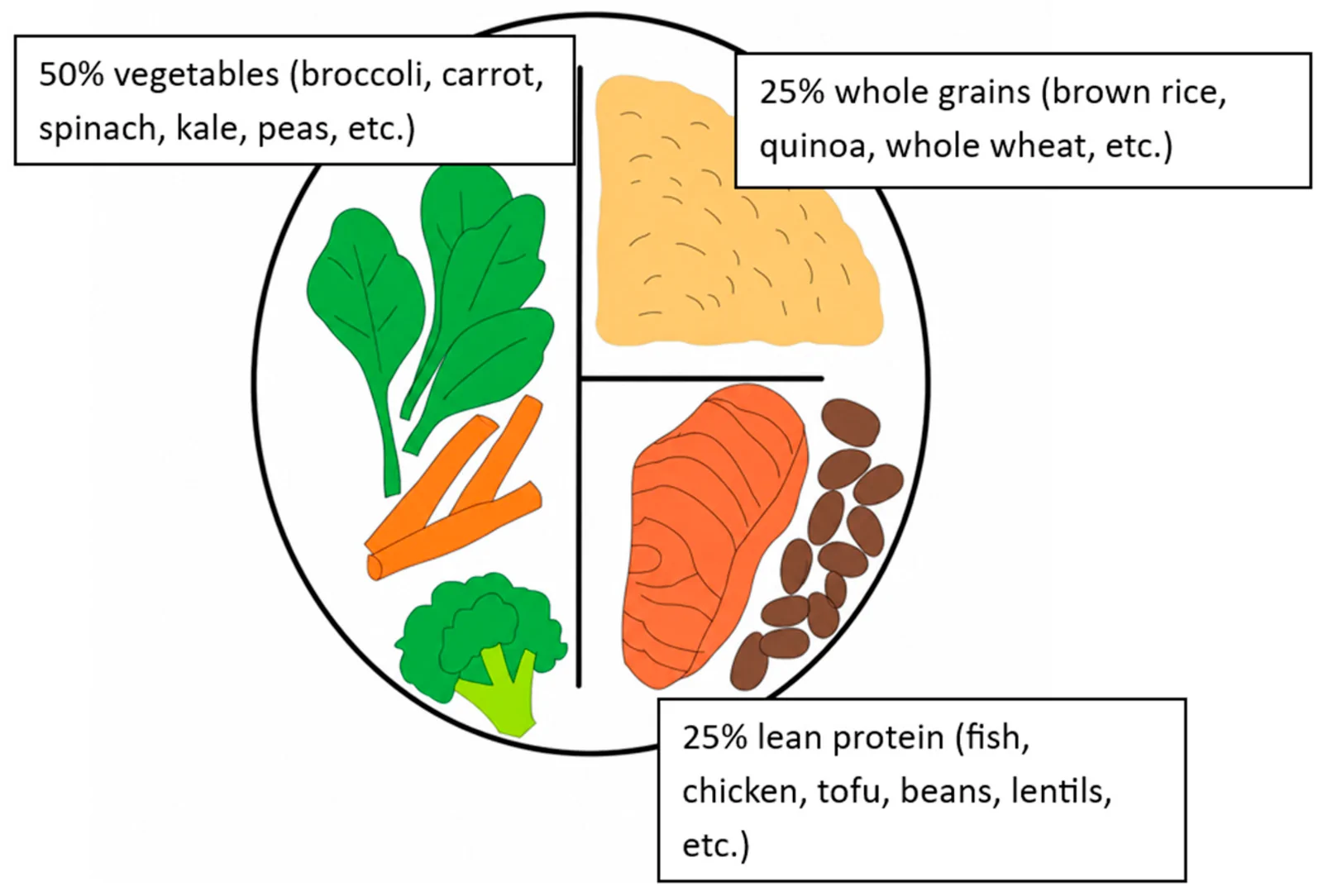
Healthy plate. The authors drew the figure.

Overall, in children with CKD and preserved kidney function, nutrition should be based on a healthy, balanced diet and, if possible, breastfeeding in infants, while avoiding unnecessary restrictions. Usually, four groups of nutrients make up a meal: starchy carbohydrates, fruits and vegetables, protein foods, and dairy products, which should be balanced and are typically recommended to consist of 50% carbohydrates, 15–20% protein, and 30–35% fat [11]. In practice, adherence is increased by simplifying recommendations for children and parents—25% carbohydrates, 25% protein, and 50% vegetables—as shown in Figure 1.

Processed foods are discouraged not only due to increased salt, as emphasized in the aspect of early kidney health, but also due to increased phosphate and potassium load [11].

**Figure 2:**
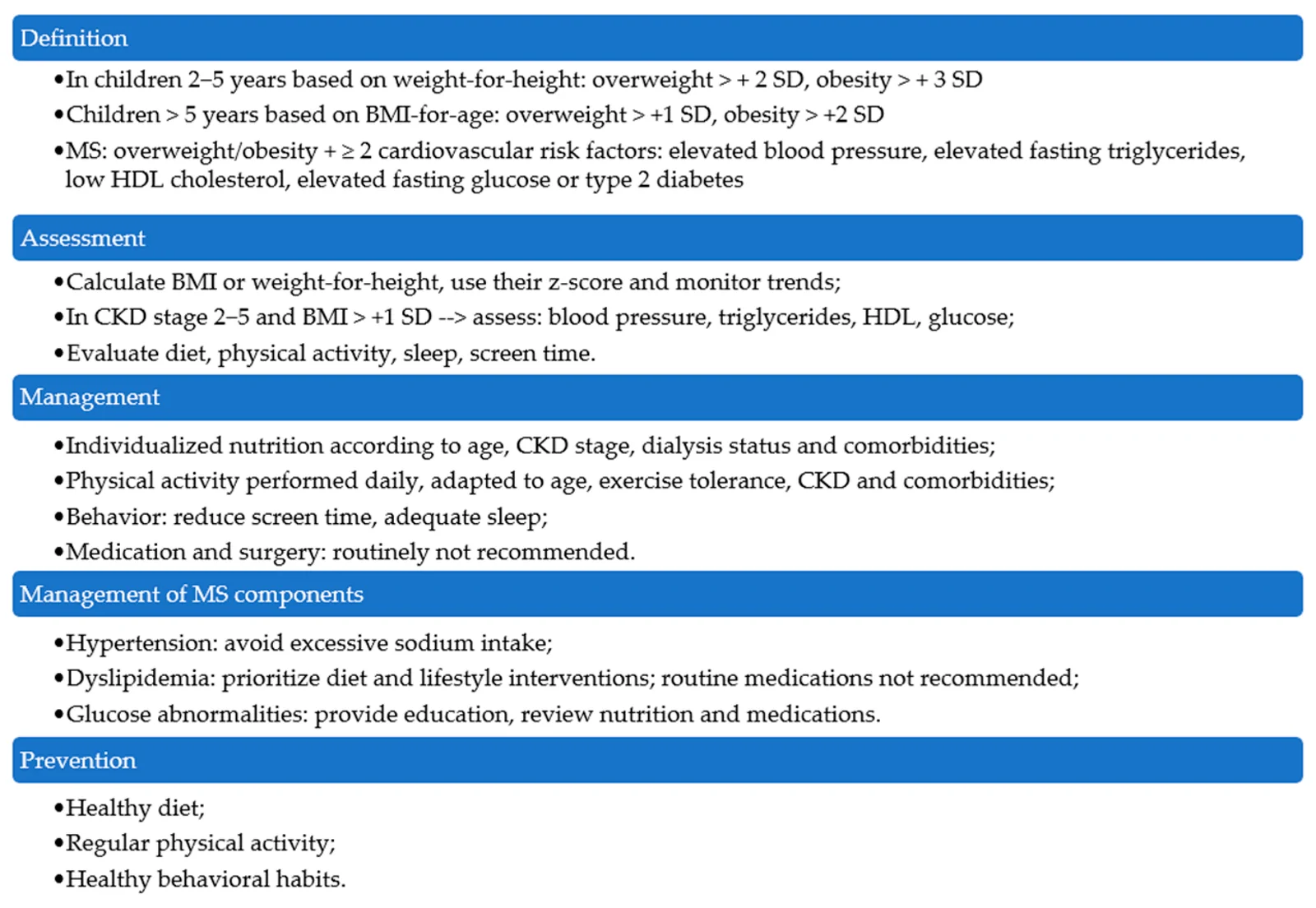
Clinical practice recommendations from the Pediatric Renal Nutrition Taskforce on obesity and metabolic syndrome in children with CKD stages 2–5; SD—standard deviation; MS—metabolic syndrome; HDL—high-density lipoprotein; CKD—chronic kidney disease.

These recommendations are also applied to children with CKD and obesity, which, as a global epidemic challenge [26], also affects children with CKD. PRNT emphasizes early identification and regular monitoring of excess weight and metabolic risk in children with CKD, followed by an individualized diet, physical activity, and behavioral intervention. Management should balance weight control with the child’s nutritional requirements and CKD-related needs. A summary of PRNT’s clinical recommendations is presented schematically in Figure 2 [6].

Children with obesity demonstrated hyperfiltration and increased microalbuminuria in obese children even without known CKD [26], demonstrating a synergistic harmful effect of obesity in children when CKD is already present. Sedentary lifestyle, inappropriate diet, and lack of aerobic exercise remain the leading behavioral styles predisposing to obesity development [27], and have led to the development of numerous approaches to address these issues, based on family-centered lifestyle modifications (focusing on nutritional improvements, physical activity, and behavioral changes) and integrating local and global policies [27,28]. In growing children, our primary goal is often to maintain their current weight as they grow, rather than strict weight loss; however, this goal depends on age, degree of obesity, growth trajectory, comorbidities, and developmental stage [29]. Basic approaches to obesity prevention and treatment are summarized in Table 2 [29]. KDIGO 2024 recommendations that children with CKD undertake physical activity are consistent with World Health Organization recommendations and aim for a healthy weight [12]. Not only does physical activity influence obesity prevention, but it also improves cardiovascular health, muscle mass, functional status, and quality of life [12].

In persistent obesity, a holistic approach to cardiovascular risk stratification should be undertaken in a tertiary center to evaluate and manage the various aspects of obesity and its comorbidities [30]. The interplay between obesity, cardiovascular risk, metabolic disturbances, and kidney health has been recognized as a single entity described by the American Heart Association and defined as cardiovascular–kidney–metabolic (CKM) syndrome recently [31]; it is rapidly gaining recognition in both adult and pediatric populations [32], again emphasizing the vulnerability of the kidneys in a state of obesity.

## 6. Nutrition in Pediatric Chronic Kidney Disease and Undernutrition with Electrolyte Imbalance

With the disease progression, obesity still significantly influences kidney status; however, due to several factors, including decreased appetite (anorexia), altered gastrointestinal motility (gastroparesis), malabsorption, chronic inflammatory conditions, intestinal dysbiosis, and uremic abnormalities of energy, protein, fat, and carbohydrate metabolism [17], the risk for undernutrition increases. Children with CKD requiring renal replacement therapy, especially dialysis, are frequently malnourished [33], and special individual dietary management is needed with increasing challenges as the disease progresses.

In children with advanced CKD, dietary adjustments are a major challenge, requiring continuous assessment of nutritional status and ongoing adjustment of the nutritional plan according to the child’s age and CKD progression. Effective nutritional support requires a coordinated team effort, with the clinical dietitian playing a key role. Achieving a balance between energy needs and protein and micronutrient intake is complicated, especially due to the many dietary restrictions. However, with appropriate counseling, optimal growth and development of children with CKD can be effectively achieved. PRNT clinical practice recommendations (covering nutritional assessment, energy and protein requirements, potassium, calcium/phosphate, fiber, vitamins, sodium, and magnesium/trace elements) and KDIGO 2024 guidance emphasize individualized nutritional management according to age, growth trajectory, CKD stage, residual kidney function, biochemical abnormalities, comorbidities, and dialysis modality [12]. KDIGO 2024 guidelines support a healthy, varied diet with more plant-based than animal-based foods and as little ultra-processed food as possible [12].

Oral feeding is the preferred route, and if limited, oral stimulation is desirable. With inadequate oral intake, supplementation should be started; in children who are unable to meet their nutritional requirements orally, supplemental or exclusive enteral feeding should be commenced [10,34] (often as a bridge to a long-term solution) and planning for gastrostomy insertion considered when long-term oral feeding is limited and, if possible, inserted along with a peritoneal dialysis catheter [34,35]. Antibiotic and fungal prophylaxis are recommended for all children undergoing gastrostomy placement, especially those who already have an established peritoneal dialysis catheter or will receive one at the same time [34]. Following the procedure, the feedings should be gradual. Continuous infusion feeding might be beneficial if vomiting is a problem. Tube feeding may be exclusive or supplemental to oral feeding; if the child develops interest, oral feeding is allowed, but attention must be paid to adequate nutrient intake [34].

Enteral feeding should be determined through partnership between medical staff and caregivers [34]. A dietitian often supports the handling of both types of feeding. Very rarely is parenteral nutrition needed, most often in conjunction with other complications. Strict attention is needed to care for exit sites of the gastrostomy and peritoneal dialysis catheter [34].

### 6.1. Fluid Balance

Fluid and electrolyte requirements vary in children depending on their primary kidney disease, on residual renal function, and, in children with chronic kidney failure, also on the method of replacement therapy. In all cases, fluid intake must be carefully assessed and balanced. Fluid, sodium, and potassium supplementation or restriction is individualized and depends on urine output, the ability to concentrate urine, hydration status, and the presence or absence of hypertension or hyperkalemia. Fluid management is influenced by residual kidney function, urine output, dialysis modality, blood pressure, intradialytic weight gain, hydration status, and sodium balance. Sodium excess can contribute to hypertension and fluid overload [36].

### 6.2. Energy and Protein Intake

Children with CKD often experience stunted growth due to protein and energy wasting and micronutrient deficiencies. In addition to growth impairment, malnutrition increases the risk of morbidity, mortality, and overall disease burden [11,18].

For this purpose, we use the Kidney Disease Outcomes Quality Initiative (KDOQI) 2008 guidelines and PRNT practice recommendations, which state that the energy needs of children with CKD are the same as those of healthy children of the same age, or 100% of the recommended (suggested) daily intake (RDI), and adjusted appropriately with overweight [10,37]. The estimated needs can be adjusted based on growth trends and weight gain. In patients who are stunted, the energy intake is increased to 120–140% of the RDI [16].

For infants, breast milk is the preferred food [10,11]. If this is not possible, use standard infant formula, or if lower potassium and/or phosphorus intake is needed, use a formula with lower potassium and/or phosphorus. PRNT recommends the use of whey-dominant formulas, with attention to fluid balance as needed, and the introduction of solid foods as recommended for healthy infants [10]. To increase energy density, breast milk or formula is supplemented with glucose polymers, fats, and carbohydrates. Concentrating formula with a higher ratio of powdered formula to water is not recommended, as this also increases the electrolyte content. For older children, add pediatric oral nutritional supplements or adult renal oral nutritional supplements to the diet [11,13]. Additionally, energy contributions from dialysate in peritoneal dialysis should not be overlooked as they amount to 9 ± 4 kcal/kg/day, especially in overweight patients. Still, there is a wide variation between patients due to differences in dialysate glucose concentration, number of cycles, dwell times, and peritoneal membrane transporter status [11].

Adequate protein intake should be considered to maintain growth and nutritional status, and the relationship between dietary protein intake and phosphorus load, which significantly impacts cardiovascular disease in children with CKD, should be taken into account [5]. Excessive protein intake can lead to the accumulation of nitrogenous waste products, resulting in uremia [14]. However, protein restriction in children with CKD is generally not recommended [5,11,17], and protein intake should be maintained at the upper limit of the RDI [11]. The 2008 KDOQI guidelines recommend providing 100–140% of the RDI of protein for ideal body weight for children with CKD stage 3. Children with CKD stages 4 and 5 require 100–120% of the RDI of protein for ideal body weight [37]. The KDIGO 2024 guidelines suggest a daily protein intake of 0.8 g/kg body weight/day in children with CKD stages 3–5 [12]. With progressive CKD, intake >1.3 g/kg/day should be avoided. They also suggest that for selected patients at high risk of kidney failure, an intake of 0.3–0.4 g/kg/day may be used under strict supervision, combined with essential amino acids and keto-analogs [12]. PRNT recommendations suggest that the target protein intake is at the upper end of the RDI and might be higher in children on dialysis [10]. Additional losses of protein must therefore be taken into account when calculating needs in children with significant proteinuria [17], in patients undergoing hemodialysis who require an additional 0.1 g/kg/day of protein, and in patients undergoing peritoneal dialysis who require an additional 0.2–0.3 g/kg/day [37]. However, in children with persisting high blood urea levels, protein intake may be adjusted towards the lower end of the RDI, after excluding other causes [10]. Therefore, current practice tends toward individualization according to CKD stage and comorbidities, and relies on individual nutrition assessment and protein intake prescription.

Inclusion of fiber in the diet is also important in children with CKD, endorsed by PRNT [38], especially with concomitant obesity; however, other benefits of fiber ingestion are also beneficial, namely, prevention of constipation, fewer gastrointestinal symptoms, reduced inflammatory state, decreased production of gut-derived uremic toxins, and reduced protein content if needed in cases of rising blood urea levels. Fiber intake should approximate that of healthy children of the same age, and should include plant-based options where possible [11,38], with a recommended total fiber intake of 14 g/1000 kcal/day for children over 1 year of age [5]. However, according to PRNT, energy and protein requirements should be prioritized over fiber requirements, particularly for those who need higher energy intake [38].

### 6.3. Lipid Intake

Dyslipidemia is a frequent complication, reported in as many as 44% of children with mild to moderate CKD, most commonly hypertriglyceridemia. In these children, less than 25% to 30% of calories should come from dietary fat along with therapeutic lifestyle modifications. Dietary fiber also reduces cholesterol levels [5].

### 6.4. Sodium Regulation

While sodium intake is generally restricted in adults with CKD, the recommendations for children with CKD are different, as many have congenital malformations of the urinary tract that cause sodium loss and therefore require additional intake. In some children, sodium intake should still be restricted, according to the KDOQI recommendations, to less than 1500–2400 mg/day [16,37]. KDIGO 2024 guidelines set the goal of less than 2 g of sodium per day, which corresponds to approximately <5 g of salt/day [12]. PRNT suggests a 24-h dietary recall or food-frequency questionnaire to identify the sodium source. It can come from non-food sources, such as medications and dialysis. Conversely, kidney disease with renal wasting as a characteristic can cause sodium depletion. In those cases, sodium should be increased, as in significant extrarenal sodium losses, hypotension, symptomatic hypovolemia, sodium-wasting-related growth failure, or substantial sodium losses during peritoneal dialysis. In the latter case, the dialysis prescription may be adjusted [23]. Strict sodium restriction should be indicated in children with CKD and blood pressure above the 50th percentile, albuminuria, fluid restriction, or corticosteroid treatment [23]. PRNT also advises starting education early, reducing processed foods, and favoring a balanced diet. Salt substitutes should be avoided in children with impaired kidney function [23]. Ways to reduce sodium intake are presented in Table 3 [13].

### 6.5. Potassium Regulation

Since hypokalemia or hyperkalemia are common in patients with CKD and can cause various secondary complications, it is important to adjust the diet to alleviate potassium imbalances [16,39]. For hyperkalemia, KDOQI guidelines recommend limiting potassium intake to 40–120 mg/kg/day for infants and young children and to 30–40 mg/kg/day for older children [37]. In contrast, KIDGO 2024 guidelines suggest that routine restriction for everyone is not recommended. Intake should be adjusted based on laboratory values, the stage of CKD, and other conditions [12]. Breast milk is naturally low in potassium and is the best diet for infants with CKD. If not available, low-potassium renal formulas should be used. If necessary, formulas may be pretreated with a potassium binder [17]. In some patients, concomitant medications are used to control serum potassium levels better [16].

PRNT recommends assessing dietary potassium in those with a potassium imbalance, as a dietary history may not accurately reflect potassium intake. In those cases, non-dietary causes should be identified, and if they are excluded, a nutritional assessment using a 24-h diet history, or a food frequency questionnaire might help identify potassium-rich foods and appropriate dietary adjustments. When dietary measures are unsuccessful, a potassium binder may be considered. Severe, life-threatening hyperkalemia requires immediate medical interventions and discontinuation of all sources of potassium [39]. In cases of hypokalemia, potassium-rich foods are encouraged; supplementation with oral medication is rarely needed [39]. Dietary management is adjusted to the individual child and their needs and guided by a trained dietitian.

### 6.6. Phosphorus Regulation

Since hyperphosphatemia or hyperparathyroidism often occur in children with CKD, we must limit phosphorus intake to prevent the occurrence of cardiovascular diseases and disorders in bone mineralization [13,40,41]. The main dietary source of phosphorus is protein; therefore, we must adopt a balanced, sensible dietary approach to ensure sufficient protein intake [17]. According to PRNT, the assessment of phosphorus (as well as calcium, discussed later) intake should be based on a 24-h diet history for healthy children and those with CKD 2–5, including additives and processed foods. Phosphorus content and bioavailability vary among foods, and phosphate additives in processed foods may be highly absorbable. When detailed information is required, a 3-day prospective diet diary/food intake record may be used. Children with CKD should be regularly assessed for total phosphorus (and calcium) and dietary sources of phosphorus identified where possible [42].

For elevated parathyroid hormone levels, KDOQI guidelines recommend limiting phosphorus intake to 100% of the age-specific RDI. For elevated PTH and serum phosphorus levels, limit phosphorus intake to 80% of the RDI [37]. Similar to potassium, the KDIGO 2024 guidelines suggest an individualized approach to phosphorus restriction, emphasizing that food choices and reducing sources of phosphate additives are particularly important when phosphorus levels are elevated [12]. PRNT suggests the intake of phosphorus within the RDI for age, without compromising adequate nutrition, enabling one to maintain serum phosphorus within the age-appropriate normal range [42]. Changes in management should be based on trends of serial results rather than a single result, considering all associated laboratory results (calcium, parathyroid hormone, vitamin D, alkaline phosphatase). In children with persistent hypophosphatemia, dietary phosphorus intake should be increased, and supplements may be necessary in some cases (e.g., with intensive dialysis or renal phosphorus wasting). Children with CKD and hyperphosphatemia or hyperparathyroidism will require further phosphorus restriction and often require phosphorus binders [42].

Breast milk is naturally low in phosphorus and is the preferred diet for infants with CKD. If breast milk is not available, a low-phosphorus renal formula is used. Both breast milk and formulas can be additionally treated with a phosphate binder [17]. It is important to educate the family about foods with higher phosphorus content, such as dairy products, meats, and legumes [16]. Avoid processed foods, as phosphorus additives are much better absorbed than naturally occurring phosphorus in foods. Healthy low-phosphorus choices for children with CKD include fruits and vegetables, as well as corn and rice [17]. To help control serum phosphate levels, phosphate binders may be prescribed, which should be taken with food. When choosing a phosphate binder, the binder’s calcium content should be considered to prevent vascular calcification [13].

### 6.7. Calcium Regulation

Calcium intake management is also challenging in children with CKD, as insufficient calcium intake leads to deficient bone mineralization, further leading to an increased risk of mineral and bone disorders in children with CKD [43]. Calcium overload increases the risk of cardiovascular disease [11]. Current KDOQI recommendations suggest that total calcium intake should be 100–200% of the age-specific RDI, or a maximum of 2500 mg of elemental calcium per day in older children [11,37]. PRNT suggests regular calcium assessment. In special circumstances (e.g., mineral-depleted bone), a higher intake may be considered with careful monitoring. Still, otherwise, in children with CKD, the recommended calcium intake is within the RDI for age, without compromising adequate nutrition [42]. Intake should be adjusted to maintain normal serum calcium, considering laboratory trends and associated laboratory results [42].

When calculating total calcium intake, total calcium exposure from food, supplements, and calcium-containing phosphate binders should be considered together [13,37]. Also, in cases of hypocalcemia, additional calcium can be provided using high-calcium dialysate [11]. Along with calcium regulation, maintaining vitamin D status is central to dietary management of calcium [5].

In infants, the only sources of calcium are breast milk or infant formula; later, milk and dairy products account for 44–70% of total calcium intake. After milk and dairy products, cereals are the most important source of calcium. Patients with CKD who do not reach the RDI require calcium supplementation [14]. According to PRNT, calcium intake above 200% of the RDI is needed in children with persistent hypocalcemia or elevated parathyroid hormone [42]. Calcium absorption in the intestine can be increased by vitamin D supplementation, which is often deficient in these patients [44].

Severe hypercalcemia can be life-endangering and requires immediate medical intervention. In cases of mild to moderate persistent cases, a stepwise approach is suggested, including reducing calcium and vitamin D supplements, calcium-based phosphate binders, and using lower-calcium dialysate [42].

### 6.8. Micronutrient Supplementation

To meet nutritional requirements in a growing child, it is important to pay attention to vitamins, minerals, and trace elements, although information on requirements in children with CKD remains limited.

PRNT assessed the management of magnesium, vitamins, and trace elements in children with CKD. Assessed requirements are generally similar to those for healthy children, with breast milk or formula and a balanced diet later providing adequate amounts of the listed nutrients [45,46]. Magnesium should be assessed when hypokalemia, hypocalcemia, symptoms/signs, or risk factors for abnormal magnesium levels are present; trace elements are not routinely checked but may be when there is suspicion of deficiency. Interactions with medications and other vitamins and trace elements should be considered, and clinical signs of excess/deficiency should be monitored [46]. With biochemical abnormalities present, a nutritional overview should be conducted, with optimization of nutrition and supplementation if unsuccessful, especially with magnesium, divided into 2–3 doses/day to reduce side effects. Again, management should be based on serial trends, rather than a single measurement [46].

In the early stages, vitamin requirements are similar to those of children without CKD but can shift toward depletion or excess as CKD progresses. Vitamin D should be routinely checked, as it is frequently insufficient in children with CKD and is associated with poor outcomes [11,47]. Vitamin status and other nutrients can be affected by diet, supplementation, kidney function, medications, and dialysis. Both deficiency and excess are possible. Based on nutritional status, some could also be checked and supplemented in cases of deficiency [45]. During dialysis, water-soluble vitamins, most commonly vitamins C, B6, and folate, can be lost, suggesting assessment [5,11]. Vitamin A should be assessed in cases of unexplained hypercalcemia or elevated intracranial pressure [45]. Folate and vitamin B12 are sought and prescribed when macrocytic anemia is present and biochemical evidence of insufficiency is present [45]. Vitamin A should not exceed the daily requirements [45] as its accumulation may lead to hypercalcemia and raised intracranial pressure. Routine supplementation of vitamin E and K is not necessary, unless in comorbidities, predisposing to deficiency (malabsorption) [11]. The benefits of vitamin C supplementation should be weighed against its risks due to its metabolism to oxalate [45].

One of the most common complications of CKD in children is anemia. Although it is most often the result of reduced erythropoietin production, some patients are inherently prone to iron deficiency and can develop anemia in association with both—iron and erythropoietin deficiency. Therefore, iron levels are not usually controlled by diet. Still, additional oral or intravenous iron supplementation must be provided to prevent depletion of stores and maintain target hemoglobin concentrations, guided by appropriate laboratory assessment and clinical status [16,48]. Simultaneous supplementation of iron with other trace elements, especially zinc and copper, is discouraged as they demonstrate competitive inhibition of intestinal absorption [46].

Non-dietary factors, namely CKD stage, dialysis, comorbidities, and medications, should be considered when assessing and monitoring specific nutrient status and planning nutritional interventions [11].

## 7. Nutrition in Children with Hypertension

Hypertension is a common complication of CKD, especially when associated with obesity [49]. Most commonly, the Diet Approaches to Stop Hypertension (DASH diet); the traditional Asian, plant-based; the Southern European traditional Atlantic; and the Mediterranean diets are discussed and presented briefly in Table 4 [50]. The DASH diet emphasizes foods and beverages low in sodium and saturated fat, while reducing processed meats, salty foods, and sweetened drinks. On the contrary, consumption of whole grains, fruits, vegetables, nuts, seeds, legumes, and low-fat dairy is strongly encouraged [50]. A conventional DASH diet can still provide relatively high potassium, phosphorus, and fluid, depending on food choices, and it may require modification in children with advanced CKD or electrolyte abnormalities.

In children with advanced CKD and electrolyte imbalance, it is important to be aware of studies showing that the DASH diet leads to higher intake of minerals such as potassium, magnesium, and calcium [50]. Otherwise, it remains the most supported strategy for the nutritional management of hypertension; meanwhile, other diets may benefit blood pressure, though evidence is limited. Limiting sodium intake is important for a nutritious approach to lower blood pressure in association with fluid intake [50], which can be challenging to tackle in children with CKD and other nutritional demands.

## 8. Dietary Adherence

Only a few studies have examined the success of adherence to different diets in children with CKD [51]. The most common limitations in this research aspect include the variety and complexity of the methods to assess dietary adherence and its description [51]. In a small study of 16 children with CKD, all children exceeded protein intake recommendations, 68% exceeded fat recommendations, and 63% failed to meet fiber intake requirements. Following a dietary intervention, adherence to certain recommendations improved [8]. In a study of 70 children with CKD, 50% had a phosphorus intake exceeding the dietary reference intake. When stricter recommendations were implemented, as many as 94% of children with hyperphosphatemia had excessive dietary phosphorus intake [52]. Another study of 36 children with CKD showed high sodium and protein intake, as well as poorer dietary quality: no child met the recommended intake of vegetables, while only 44% met the recommendation for fruit and 31% for grains and dairy [53]. A study of dietary intake among children in the CKiD cohort revealed that children with CKD consumed, on average, higher amounts of sodium, phosphorus, protein, and energy than recommended [54]; this indicates a significant discrepancy between recommended and actual intake, as well as the need for an individualized approach involving a trained dietitian.

Children with CKD face several challenges when adhering to dietary recommendations, such as frustration with unpalatable food, deprivation by restriction leading to rule-breaking, and compounding the burden of caregiving and clinical management. Restrictions can affect social interactions and daily routines, leading to increased frustration and occasional dietary nonadherence [55]. Consequently, cultural norms might be disrupted as the child might not be able to eat foods tied to their cultural or family customs. Also, dietary restriction can present a barrier to social engagement and participating in social activities. Caregivers are worried about children attending kindergarten, surrounded by peers who are eating (and maybe even sharing) restricted snacks. Children with CKD often feel excluded from normal experiences, and adolescents express the feeling of deprivation of normal social interactions [55].

## 9. Conclusions

The spectrum of CKD in children varies from preserved kidney function and overweight to end-stage kidney failure and undernutrition. Consequently, nutrition, a basic part of a patient’s management, should be tailored to the child’s age, growth, nutritional status, kidney function, and treatment modality. Children with preserved kidney function benefit primarily from optimizing cardiovascular health and kidney outcomes, which helps prevent obesity and other metabolic imbalances. Children with advanced CKD require an individualized approach with meticulous attention to energy, protein, lipid, fluid, electrolyte, and micronutrient intake to support normal growth and development and to minimize CKD-related complications.

Regular nutritional assessment, inclusion of the whole family, continuous education, and individualized dietary counseling are integral components of care.

**Figure 3:**
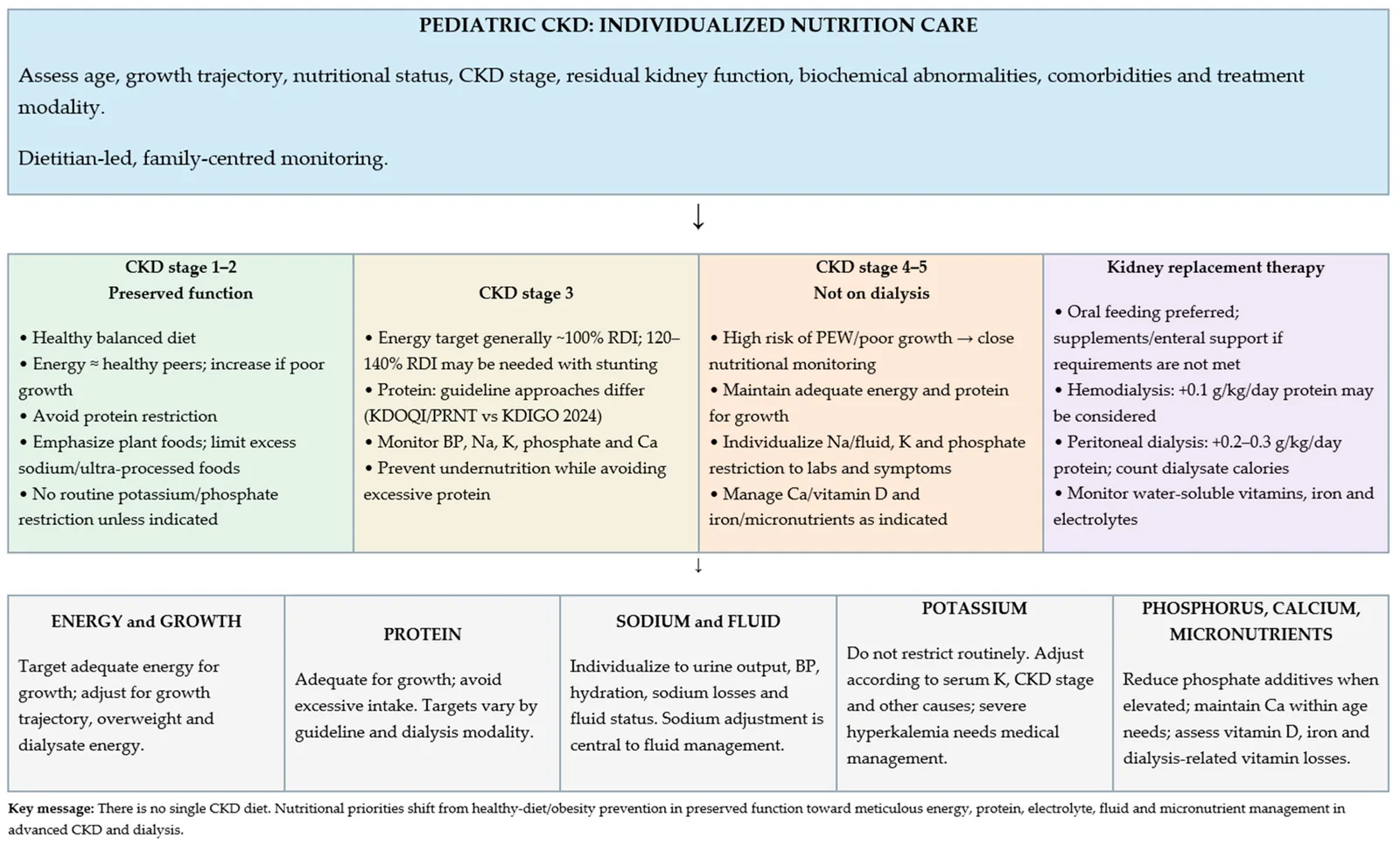
Summary of major nutritional priorities by CKD stage and treatment modality; CKD—chronic kidney disease; RDI—recommended daily intake; KDOQI—Kidney Disease Outcomes Quality Initiative; KDIGO—Kidney Disease: Improving Global Outcomes; PRNT—Pediatric Renal Nutrition Taskforce; BP—blood pressure; Na—sodium; K—potassium; Ca—calcium.

To conclude, a summary of major nutritional priorities by CKD stage and treatment modality is presented in Figure 3.

## Figures and Tables

**Table 1:** Nutritional assessment according to Pediatric Renal Nutrition Taskforce; BMI—body mass index, SDS—standard deviation score, WHO—World Health Organization, CKD—chronic kidney disease.

Parameter	Recommendation
Weight and height/length	Measure routinely in all children. Use euvolemic (“dry”) weight, particularly when fluid status is abnormal, such as in dialysis or nephrotic syndrome.
Head circumference	Measure through age 2 years (or up to 3 years when appropriate growth charts are available).
Growth charts	Plot measurements serially on WHO or appropriate national growth charts.
Growth z-scores	Calculate z-scores/SDS to complement the growth charts.
Growth velocity	Assess height/length velocity over at least 6 months; trends are important for identifying impaired growth.
BMI	Calculate BMI from age 2 years onward; for younger children use weight-for-length.
Short stature	If height is below the 3rd percentile, use height age when calculating BMI z-score, provided the child has not reached adult height.
Genetic/disorder-specific growth	Use condition-specific growth charts when available.
Prematurity	For premature infants, track growth using both gestational and chronological age for the recommended period.
Monitoring frequency	Monitor growth routinely, more frequently in younger children and those at higher nutritional/growth risk, including those with advanced CKD.
Dietary assessment	Tailor assessment to disease severity, growth, intake, gastrointestinal symptoms, and biochemical abnormalities. Assess appetite when nutritional targets aren’t being met.
Diet history	When detailed assessment is needed, use a prospective ≥3-day food diary. A 24-h recall can be used, preferably on more than one day.
Biochemical assessment	In adolescents on hemodialysis, protein catabolic rate can help assess protein adequacy, especially when followed over time.
Serum albumin	Should not be interpreted as a nutritional marker alone until non-nutritional causes of low albumin have been excluded.

**Table 2:** Basic approaches to obesity management in children.

Nutritional Adjustments	Physical Activity and Sleep	Behavioral and Family Support
Elimination of sweet drinks	At least 60 min of movement daily	Changing family habits (involvement in nutritionally and physical adjustments with all family members)
Adequate portion sizes	Active play (bike riding, family walks)	Avoidance of weight stigma
High-quality foods (whole grains, vegetables, fruits)	Limited screen time	Realistic goals
Lean proteins (poultry, fish, beans, low-fat dairy)	Age-appropriate sleep schedule	Psychological support
Avoidance of strict dieting		
Limited processed snacks		

**Table 3:** Practical suggestions to minimize sodium intake in children.

Consuming foods low in sodium (fresh fruits and vegetables, fresh meats, soft cheeses, goat cheese, snacks without added salt)	Eating freshly prepared food and minimizing the intake of industrially processed food	Checking the sodium content on food labels (salty food by definition contains more than 140–200 mg sodium per serving or 6 to 9 mmol sodium per serving)
Avoiding foods high in sodium (soy sauce, meat products (ham, bacon, sausages, hot dogs), hard cheeses, processed cheese, cheese spreads, pickled vegetables, ketchup, salted crackers, chips, salty snacks and dried soup mixes)	Replacing sodium chloride with potassium chloride, but not in children with hyperkalemia	Using spices, herbs, lemon or lime juice and vinegar instead of salt
Avoiding adding salt	Eating home-cooked food, and avoiding fast food	Limiting or avoiding the use of salt in cooking

**Table 4:** Dietary approaches in children with hypertension that yielded some favorable results in blood pressure reduction in children.

Diet/Dietary Pattern	Main Characteristics	Evidence in Children/Adolescents
Dietary approaches to stop hypertension	Emphasizes fruits, vegetables, whole grains and other healthy foods	Evidence available in children and adolescents
Mediterranean diet	Rich in fruits, vegetables, whole grains, fish, eggs, nuts and typically olive oil; high adherence is associated with a healthier overall dietary pattern	Higher adherence is associated with lower blood pressure values, although responses may vary with genetic risk
New Nordic Diet	Healthy Nordic dietary pattern based on Nordic Nutrition Recommendations	Higher achievement of dietary targets was associated with lower systolic and diastolic blood pressure
Traditional Asian diets (Japanese, Korean, Chinese)	Traditional dietary patterns characteristic of Japan, Korea and China	Evidence is more limited; less evidence is available for the traditional Chinese diet
Plant-based diets	Predominantly plant-based eating; may include vegetarian/vegan patterns	Evidence of blood pressure reduction exists, but nutritional deficiencies (particularly vitamin D, iron and B12) can occur; the source notes that vegan diets require expert supervision in children

## Data Availability

No new data were created or analyzed in this study. Data sharing is not applicable to this article.
